# Optimizing Dental Bond Strength: Insights from Comprehensive Literature Review and Future Implications for Clinical Practice

**DOI:** 10.3390/biomedicines11112995

**Published:** 2023-11-08

**Authors:** Yung-Shin Fan-Chiang, Peng-Chen Chou, Yu-Wen Hsiao, Yu-Hsuan Cheng, Yi Huang, Yu-Chieh Chiu, Yu-Ju Lin, Yuichi Mine, Sheng-Wei Feng, I-Ta Lee, Tzu-Yu Peng

**Affiliations:** 1 School of Dentistry, College of Oral Medicine, Taipei Medical University, Taipei 110, Taiwan; b202109030@tmu.edu.tw (Y.-S.F.-C.); shengwei@tmu.edu.tw (S.-W.F.); 2 Department of Medical Systems Engineering, Graduate School of Biomedical and Health Sciences, Hiroshima University, Hiroshima 734-8553, Japan; 3 Project Research Center for Integrating Digital Dentistry, Hiroshima University, Hiroshima 734-8553, Japan

**Keywords:** dental restorations, bond strength, dentin-resin bonds, resin-resin bonds, smear layers, MMP inhibitors, saliva contamination, ceramics pretreatment

## Abstract

This review examines the modifying factors affecting bond strength in various bonding scenarios, particularly their relevance to the longevity of dental restorations. Understanding these factors is crucial for improving clinical outcomes in dentistry. Data were gathered from the PubMed database, ResearchGate, and Google Scholar resources, covering studies from 1992 to 2022. The findings suggest that for dentin-resin bonds, minimizing smear layers and utilizing MMP inhibitors to prevent hybrid layer degradation are essential. In the case of resin-resin bonds, reversing blood contamination is possible, but preventing saliva contamination is more challenging, underscoring its critical importance during clinical procedures. Additionally, while pretreatment on ceramics has minimal impact on bond strength, the influence of specific colorings should be carefully considered in treatment planning. This comprehensive review highlights that although established practices recognize significant bond strength factors, ongoing research provides valuable insights to enhance the clinical experience for patients. Once confirmed through rigorous experimentation, these emerging findings should be swiftly integrated into dental practice to improve patient outcomes.

## 1. Introduction

Resin composite restorations are a cornerstone of modern dentistry known for their ability to seamlessly blend aesthetics, convenience, and mechanical properties. These restorations, which bond directly to the tooth structure, have revolutionized dental practices, offering patients durable and natural-looking solutions [1,2]. The bond strength between the resin composite and the tooth is a critical factor in ensuring the long-term success of these restorations. Weak bond strength can lead to instability between the restoration and the tooth, significantly increasing the risk of secondary caries and restoration failures [3,4,5]. 

This review embarks on a comprehensive exploration of bond strength in dentistry, with a particular focus on resin composite restorations. We will delve into the mechanisms by which resin composites bond to the tooth structure, highlighting the significance of this bond strength in maintaining the integrity of restorations. The ability to achieve and maintain a strong bond is crucial for the longevity and success of dental treatments. In addition to examining the bond strength between dentin and resin, we will also explore the bond strength between different resin composites and the bonding of resin to ceramic materials, addressing each scenario in detail [6,7]. To provide a holistic understanding, we will start by discussing various methods used to test bond strength, such as tensile bond strength testing (TBS), micro-tensile bond strength testing (µTBS), shear bond strength testing (SBS), and micro-shear bond strength testing (µSBS). We will elaborate on the advantages and limitations of these testing methods, particularly in the context of different clinical situations [8,9,10].

Furthermore, we will investigate the key factors influencing bond strength in dentistry. Surface treatment, adhesive systems, curing methods, and environmental conditions all play pivotal roles in determining the strength and durability of bonds. Understanding how these factors interact with different materials will offer valuable insights for dental practitioners and researchers alike. The review will also highlight recent advancements in dental materials and technologies, which have significantly contributed to improving bond strength in dental restorations [11,12]. This includes innovations in resin composites, adhesive systems, and bonding techniques [13,14]. Staying up-to-date with these latest developments is crucial for dental professionals to make informed decisions that enhance the quality and longevity of their treatments.

The aim of this literature review is to provide a comprehensive resource for understanding bond strength in dentistry, with a focus on resin composite restorations. By examining the mechanisms of bonding, testing methods, influencing factors, and recent advancements, we aim to equip dental professionals with the knowledge and tools needed to achieve optimal bond strength in clinical practice. This comprehensive approach ultimately leads to improved patient outcomes and long-lasting dental restorations.

## 2. Bond Strength Testing Strategy

**Figure 1:**
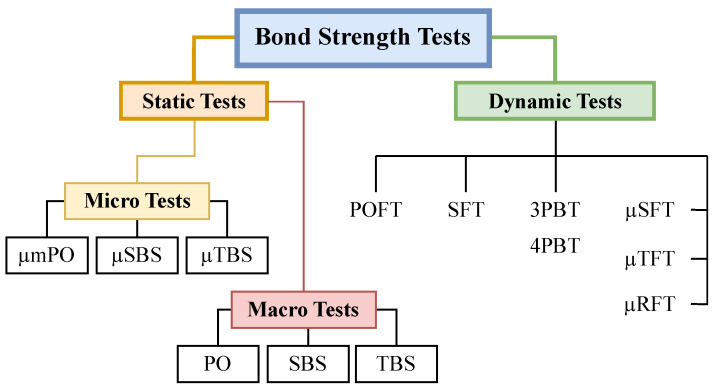
Strategy of bond strength testing used in the laboratory (µmPO, micro push-out test; µSBS, micro shear bond strength; µTBS, micro tensile bond strength; PO, push-out test; SBS, shear bond strength; TBS, tensile bond strength; POFT, push-out fatigue test; SFT, shear fatigue test; 3PBT, 3-point-bend fatigue test; 4PBT, 4-point-bend fatigue test; µTFT, micro tensile fatigue resistance test; µSFT, micro shear fatigue resistance test; µRFT, micro rotary fatigue test).

Within the laboratory, a wide range of bond strength tests exists. These tests can be categorized into two main groups: static tests and dynamic tests, which differ based on whether the sample is fixed during testing (Figure 1). In the static tests category, macro tests assess bond areas larger than 3 mm^2^, while micro tests focus on bond areas around 1 mm^2^ [15]. Both macro and micro tests share similar subcategories, including SBS, µSBS, TBS, µTBS, and push-out tests (PO) [8]. Static bond strength tests are often considered less clinically significant as they do not accurately replicate realistic situations. Dynamic tests are generally preferred. However, Poitevin et al. [16] demonstrated that the µTBS test may actually be a more reliable method than the micro tensile fatigue resistance test (µTFR).

### 2.1. Dynamic Tests

Long-term bonding effectiveness is primarily assessed through intricate and labor-intensive dynamic tests, resulting in fewer fatigue test studies. These tests are crucial for evaluating how dental restorations can endure real-world stress over time, vital for gauging adhesive system longevity. Notably, assessing enamel bond fatigue presents distinct challenges, with recent research predominantly emphasizing dentin bonding dynamics [17,18]. Dynamic tests, such as the POFT, simulate repetitive masticatory forces, helping researchers gauge the durability of the bond between restorative materials and teeth [19,20]. The SFT assesses an adhesive system’s ability to withstand shear forces, particularly relevant in posterior teeth during chewing [21]. The 3PBT and 4PBT tests replicate flexural stresses, providing insights into dental restoration performance during activities like chewing [22,23]. Recent research increasingly focuses on micro-scale dynamic tests like the µTFT, µSFT, and µRFT, which offer detailed microscopic assessments for applications like veneers and inlays [16,24]. While dynamic tests are more challenging and time-intensive than static tests, they offer vital insights into the long-term durability of adhesive systems. Striking a balance in research between enamel and dentin bond fatigue studies is crucial for a comprehensive grasp of bonding longevity and the ongoing enhancement of dental restoration materials and techniques [25,26,27].

### 2.2. Static Tests

In the previous century, macro test methods were dominant in material testing until micro tests emerged, offering valuable insights despite their complexity. It is worth noting that evaluating bond strength alone may not entirely reflect the clinical performance of adhesive systems, emphasizing the necessity of incorporating them into a broader range of experiments for a more comprehensive understanding [7,28,29]. Material testing encompasses the essential PO test which assesses interfacial strength and bonding quality [30]. Tensile bond strength tests are equally vital for evaluating an adhesive system’s resistance to being pulled apart, especially in dental applications facing various mechanical stresses [31]. In recent years, micro test methods have gained prominence, including µmPO, which examines micro-level bond strength, offering insights into smaller, intricate dental applications like inlays and onlays [32]. The µSBS explores resistance to shearing forces at the micro-level, while µTBS assesses micro-level performance with precision, aiding delicate procedures like veneer applications [33,34]. These tests provide a comprehensive understanding of adhesive system performance. While the tests mentioned provide insights into the static properties of adhesive systems, it is important to note that clinical performance is not solely dependent on bond strength. To gain a comprehensive understanding of their real-world functionality, it is crucial to combine bond strength tests with other assessments. Microleakage studies evaluate the system’s ability to prevent fluid and bacterial ingress at restoration margins, critical for restoration longevity. Wear resistance tests assess how well the system withstands everyday oral abrasion and friction. Clinical trials play a vital role in evaluating real patient scenarios and long-term effectiveness. Integrating bond strength tests with these complementary assessments provides a more clinically relevant perspective, enhancing evaluation accuracy and alignment with real-world applications [29,35].

## 3. Dentin-Resin Bonding

### 3.1. Smear Layer

The smear layer is an organic layer encountered during endodontic treatment and root canal cleaning. It is a homogeneous and amorphous structure that appears to completely close off the dentinal orifices, which, under most circumstances, should be removed [36,37,38]. Due to the micro-porous structure of the dentin tubules, fluids from the dentin tubules can pass through the smear layer. However, bonding resin is hydrophobic, so it cannot penetrate such a debris-laden area. The smear layer is usually removed prior to the application of adhesive [39]. Different surface preparations can lead to different pre-processing results [40,41]. Both the smoothness of the surface and the thickness of the smear layer will affect the bonding force between the subsequent resin and dentin. Resin cements are also an impacting factor on bond strength [42]. 

The following are some of the most commonly used and discussed tooth preparation instruments: silicon carbide (SiC) abrasive grinding paper (P120 grit, P400, P1200 grits), ground diamond bur, diamond bur, carbide bur, and superfine-grit bur [43]. Results have shown that bond strength is higher when dentin is prepared with a superfine-grit bur, as the surface demonstrates higher bond strength compared to the regular-grit group. Out of the four kinds of burs tested, preparation carried out by the carbide bur showed the weakest bond strength. Diamond burs produce a denser smear layer compared to diamond burs ground with SiC abrasive grinding paper.

Resin cements such as RelyX Ultimate (3M ESPE, St Paul, MN, USA), RelyX ARC (3M ESPE, St Paul, MN, USA), RelyX U200 (3M ESPE, St Paul, MN, USA), Multilink Automix (Ivoclar Vivadent AG, Schaan, Liechtenstein), and Panavia V5 (Kuraray Noritake Dental Inc., Tokyo, Japan) are common choices in the field of dentistry. This research also suggests that multi-laminate carbide steel burs produce a thin and more regular smear layer, which is ideal [44]. Another group of researchers found that Panavia V5 (Kuraray Noritake Dental Inc., Tokyo, Japan) showed higher bond strengths than other resin cements, and RelyX ARC (3M ESPE, St Paul, MN, USA) achieved higher bond strength values compared to RelyX U200 (3M ESPE, St Paul, MN, USA) [45]. It is worth noting that, under special treatment, certain results can be achieved. By combining Panavia V5 (Kuraray Noritake Dental Inc., Tokyo, Japan) with a superfine-grit bur and applying it twice, the highest bond strengths were obtained. Additionally, a double application of primer improved the bond strength of all cements to dentin [46].

The application of phosphoric acid is a common method to remove the smear layer. Lower concentrations of phosphoric acid exhibit weaker etching effects, failing to completely eliminate the smear layer, while higher concentrations result in poor demineralization and low bond strength [47]. In recent years, attention has turned to weak organic acids like polyacrylic acid (PAA) for surface conditioning [48]. PAA provides an alternative means to modify substrate surfaces. When applied to dental surfaces, PAA can alter surface chemistry and morphology, preparing them for bonding with resin materials. Lower concentrations of PAA may offer milder etching compared to phosphoric acid, presenting a gentler surface conditioning option. However, higher PAA concentrations can lead to poor demineralization, emphasizing the need for precise concentration selection. The effectiveness of surface conditioning is influenced by various factors, including the choice of acid, surface smoothness, and smear layer thickness. Dentists must consider these variables when selecting the most suitable acid and concentration for specific procedures [48]. Furthermore, comparing smear layer treatments, it has been noted that a diamond turning needle generates a denser smear layer compared to surfaces ground with SiC abrasive grinding paper. This distinction in smear layer characteristics can significantly impact subsequent bonding, underlining the necessity for a customized approach to surface conditioning in dentistry. Dentists must carefully assess surface conditions and method selection to achieve optimal bonding results [49,50].

### 3.2. Matrix Metalloproteinase (MMP)

The endogenous protease matrix metalloproteinase (MMP) is a group of host-derived proteolytic enzymes that depend on calcium and zinc. These enzymes are entrapped within the mineralized dentin matrix. MMPs hydrolyze the organic matrix of demineralized dentin, leading to the degradation of exposed collagen fibrils beneath the hybrid layer and significantly weakening the bond strength between resin and dentin. MMPs play various roles in dentistry, including dentinogenesis, caries progression, and bonding stability [51,52,53]. They encompass three domains: a catalytic prodomain with a zinc-binding site, a hinge region, and a hemopexin domain. Consequently, the activation of MMPs necessitates four factors: prodomain cleavage, dependence on a zinc ion, the preservation of specific amino acid sequences, and the inhibition of enzyme activity by tissue inhibitors of metalloproteinases (TIMP).

### 3.3. Effects on MMP by Simplified Adhesive

Within MMPs, a cysteine-switch mechanism can be initiated in a low pH environment, consequently leading to the activation of these enzymes by inducing conformational changes in their properties. It is noteworthy that the maximum catabolic activity of MMPs is achieved under acidic conditions, typically within the pH range of 2.3 to 4.5, and the process of neutralization can significantly augment MMP activation. This acidic milieu can be attributed to various sources, including the production of lactate by cariogenic bacteria and the presence of mild acids in both etch-and-rinse adhesives and self-etching adhesives. The activation of MMPs within the hybrid layer entails a two-step process. Initially, there is an upsurge in MMP activity due to the demineralization of dentin. Subsequently, there is an additional increment in activity triggered by the exposure of collagen. It is imperative to note that while a low pH environment is essential for MMP activation, it is preferable to employ mild acids rather than strong acids. The use of strong acids can lead to the denaturation of the enzymes. In this regard, many simplified adhesives incorporate acidic monomers with a pH ranging from 1 to 2. These mild acids effectively demineralize dentin without reaching acidity levels sufficient to denature MMPs. Consequently, the utilization of simplified adhesives may result in the gradual degradation of exposed collagen at the bonding interface, leading to a progressive reduction in the strength of resin-dentin bonding over time [52,53,54].

### 3.4. MMP Inhibitors

Inhibiting MMP activity plays a pivotal role in maintaining the strength of resin-dentin bonds. In contemporary dentistry, numerous bonding systems integrate MMP inhibitors, contributing to the extended durability of resin-based restorations. There exist three fundamental principles for MMP inhibition: chelation of calcium or substitution of zinc ions, thereby obstructing MMP access, surface coating of the substrates, and the use of cross-linking agents. Cross-linking agents have gained significant attention as an innovative approach to inhibiting MMPs and enhancing the longevity of resin-dentin bonds. These agents work by promoting the formation of additional chemical bonds within the dentin matrix [55]. The process involves creating covalent links between collagen molecules, effectively reinforcing the collagen network. This reinforcement not only strengthens the dentin structure but also makes it more resistant to enzymatic degradation by MMPs. The use of cross-linking agents is particularly effective in stabilizing the hybrid layer, where resin materials interact with dentin [56]. In this critical interface, the application of cross-linking agents helps to lock the collagen fibers in place, preventing them from being broken down by MMPs. This enhanced stability contributes to improved bond strength and, ultimately, the longevity of dental restorations [57]. The subsequent list outlines some commonly used MMP inhibitors [52,53,54,58,59]:

1. TIMPs
2. Quaternary ammonium methacrylates (QAM)
3. Collagen cross-linkers (ex. ethylenediaminetetraacetic acid (EDTA), galardin, chitosan, riboflavin, etc.) [53,58,59]
4. Protease inhibitors
5. Chlorhexidine (CHX) [52,54]
6. Tetracyclines and non-antimicrobial chemically modified tetracyclines (CMTs).

Previous researchers have conducted extensive investigations into the mechanisms of the MMP inhibitors mentioned above [60]. Among these inhibitors, TIMPs are known to reversibly inhibit MMPs. Notably, TIMP-3 demonstrates superior efficacy in inhibiting MMP-9 when compared to other TIMPs. MMP-9 is closely associated with a reduction in dentin-resin bonding strength. CHX functions as a non-specific MMP inhibitor by binding to calcium and zinc ions through chelation, reducing the degradation of the hybrid layer without adversely affecting bonding strength. However, it should be noted that CHX does not necessarily improve bond durability. The CMTs exhibit the capability to chelate zinc ions and down-regulate MMP mRNA expression. They disrupt the protein activation process, rendering MMPs more susceptible to hydrolysis. Riboflavin serves as a collagen cross-linking agent, typically used in conjunction with UV light. It exerts biological inhibition on dentin MMPs, particularly MMP-9. In addition to inhibiting MMP activity, riboflavin enhances bond strength and stability by improving the mechanical properties of dentin collagen fibers and making them more resistant to hydrolysis. However, it is worth noting that the safety of riboflavin in dentistry remains a topic of ongoing debate. EDTA serves as a calcium ion chelator and can enhance the hybrid layer’s resistance to degradation when used after etching, especially when employed in conjunction with the ethanol wet-bonding technique to achieve greater bond strength. However, EDTA’s acid etching effect is relatively slow, and it may result in a bond with dentin that is too loose for effective adhesion. In the context of dental caries progression, both MMPs and cysteine cathepsins are involved, including collagenases (MMP-1, MMP-8), gelatinases (MMP-2, MMP-9), stromelysin (MMP-3), and enamelysin (MMP-20). Additionally, saliva and dentinal fluid also contain various MMPs and cathepsins [53]. Among these enzymes, MMP-2 and MMP-9 exert the most significant influence on the caries process [61]. Furthermore, several other MMPs play substantial roles in a wide range of physiological and pathological processes [62].

### 3.5. Hybrid Layer and Its Degradation

The hybrid layer is formed when resin infiltrates the dentin and undergoes polymerization due to the presence of its hydrophilic and hydrophobic chemical groups. This process results in the creation of a transitional “hybrid” layer, which is of paramount importance in dentin bonding. Generally, a thicker and more uniform hybrid layer within the normal range contributes to superior bond strength. This layer effectively seals the surface, preventing leakage, and provides robust resistance to acidic environments. The ratio of hydrophilic to hydrophobic chemical groups in the resin also plays a significant role in adhesion. An increase in resin hydrophilicity is correlated with higher bond strength. Consequently, dentin adhesives that exhibit high hydrophilicity or absorb excessive amounts of water tend to experience a reduction in stiffness. Conversely, if adhesives are overly hydrophobic or dry, it can lead to the collapse of exposed collagen fibers, resulting in weaker bonding strength [63]. Notably, a separate study indicates that wet bonding of dentin with ethanol can yield bond strengths even higher than those achieved with the most hydrophilic resins, suggesting that wet bonding with ethanol may enhance bond strengths further [64]. Over time, the mineral phase within dentin gradually depletes, leading to the exposure of collagen scaffolds. Subsequently, these scaffolds may undergo hydrolysis or degradation through bacterially derived enzymes or endogenous proteases [51,52,53]. The degradation of collagen fibrils is influenced by both chemical and physical factors. Here are four common sources of chemical factors contributing to this process [54]:

1. The application of both etch-and-rinse and self-etch adhesive systems influences the bonding process.
2. Elevated MMP levels and increased activity in adhesive-treated dentin can lead to a reduced inhibitory function of TIMPs, affecting the maintenance of bond strength.
3. Saliva containing cholesterol esterase and pseudocholinesterase contributes to a decrease in bond strength. Furthermore, bacterial collagenases induce nanoleakage at the dentin-resin interface, and acids produced by cariogenic bacteria activate MMPs, resulting in a reduction in the durability of resin-dentin bonds.

### 3.6. Methods for Better Monomer Infiltration and Inhibition of Hybrid Layer Degradation

Numerous studies have demonstrated that integrating agents with anti-MMP properties, antibacterial functions, or remineralization capabilities into adhesive systems represents practical and promising approaches to prevent the degradation of the hybrid layer, consequently enhancing the strength and durability of dentin-resin bonds. Here are several methods for preventing hybrid layer degradation [51,54]:

1. Hydrophobic adhesives (application of a hydrophobic coating).
2. Application of multiple layers.
3. Extended polymerization time by lengthening the curing time.
4. Increase solvent evaporation.
5. Use of electric current that can enhance monomer infiltration in dentin.
6. Adhesive with remineralization function.
7. Antibacterial bonding system.
8. MMP inhibitors.

## 4. Resin-Resin Bond

### 4.1. Impact of Contaminations on Resin-Resin Bond Strength

Resin-based composite stands as one of the most extensively employed materials within the field of restorative dentistry today, largely owing to its highly advantageous material properties. To optimize outcomes and deter microleakage, the incremental placement of resin is often recommended as a standard practice [65]. However, this method, while beneficial in preventing microleakage, introduces the challenge of extended working times, consequently rendering contamination control more demanding. Contaminants possess the potential to interact with residual monomers and free radicals, both of which play a pivotal role in facilitating resin-resin bonding [66]. Furthermore, it is important to acknowledge that adhesive junctions attain increased strength when the adhesive and adherent materials are in closer proximity [67], or, put differently, when the bond interface experiences minimal contamination. Thus, it becomes imperative for us to gain a comprehensive understanding of the repercussions of contaminants on resin-resin bond strength and to ascertain the most efficacious approaches for restoring the bond strength of contaminated surfaces.

### 4.2. Blood

Blood contamination has been identified as a significant factor that substantially reduces the bonding strength between resin-based composite increments [68]. Nevertheless, it is noteworthy that the bond strength of various types of resin-based composites experiences a notable recovery when a blood-contaminated surface is thoroughly rinsed with water and subsequently dried. In a study conducted by Eiriksson et al. [69], it was demonstrated that adhesives containing water as either a solvent or co-solvent tend to yield weaker resin-resin bonds on rinsed surfaces. In contrast, adhesive formulations containing acetone can effectively eliminate moisture through the simultaneous evaporation of water and acetone [70], resulting in the complete removal of residual moisture [71]. Conversely, adhesives containing water are more susceptible to the presence of lingering moisture, leading to dilution and the formation of weaker bonds [72]. In a similar investigation conducted by Carneiro et al. [73], the practice of acid-etching with phosphoric acid prior to the application of a bonding agent was shown to successfully restore the resin-resin bond strength to a level comparable to the control group, without causing significant alterations in the morphological pattern of the contaminated surface [74]. This approach has proven to be effective in mitigating the adverse effects of blood contamination on the bonding strength of resin-based composites.

### 4.3. Saliva

Saliva contamination represents a significant detriment to the bonding strength between resin-based composite increments. Neither rinsing the contaminated surface with water nor utilizing forced air to remove saliva can fully reinstate the resin-resin bond strength of various resin-based composites [75]. In fact, the application of forceful drying is not recommended as an effective decontamination method. This is due to the fact that the solid components found in saliva, such as glycoprotein sugars, tend to form a smooth layer known as a saliva film on the surface. While abrasion and the use of adhesive can enhance bonding strength [76], a smooth surface, like the one formed by the saliva film, adversely impacts the bonding strength. The most dependable method for decontamination involves the application of adhesive without prior rinsing with water. Hydrophilic solvents are highly effective in displacing moisture originating from saliva [77]. Solvents such as acetone or ethanol and their derivatives can neutralize saliva contamination and restore bond strength by denaturing the glycoprotein sugars. Apart from the direct application of a bonding agent to a saliva-contaminated resin surface, studies have demonstrated that abrasion followed by etching and the application of an adhesive system can successfully restore bond strength to levels comparable to the control group on surfaces that have already been rinsed [78]. Likewise, etching followed by the application of silane and subsequent adhesion has shown the capability to restore bond strength to control levels, although the efficacy of silane application remains a subject of debate due to inconsistent experimental results [79,80].

## 5. Ceramic-Resin Bond

### 5.1. Nano Resin and Ceromer

In recent times, a groundbreaking approach to indirect dental restorations has emerged in the form of ceramic-optimized polymers, commonly referred to as ceromers. These materials have demonstrated their potential in the dental field due to significant advancements in both their physical and mechanical properties. Ceromers are frequently employed for indirect restorations, primarily because their elastic modulus closely approximates that of dentin tissue, while their wear resistance mirrors that of natural teeth [81]. Numerous investigations have provided evidence of a positive correlation between bond strength and elastic modulus in ceromers. Notably, ceromers exhibit a superior bond strength compared to resin nano-ceramic restoratives in various tests involving acid etching, sandblasting, and adhesive applications. It has been observed that hydrofluoric acid (HF) can partially dissolve the glassy phase of ceramics, enhancing the mechanical interlocking between the restoration surface and adhesive cement. This effect can pose challenges for resin bonding. Consequently, some researchers have proposed the use of neutralizing agents as a potential solution to this issue [82]. On the contrary, other studies have cast doubt on the effectiveness of the neutralizing process in reinforcing the bond strength between resin and ceramics. Thus, the application of a neutralizing chemical to the ceramic surface after etching remains a subject of debate in the field of dental restorative procedures.

### 5.2. Surface Pretreatment 

In a study conducted by Lee et al. [83], the researchers investigated the impact of various pretreatments on the bond strength between glass-infiltrated alumina and different resin materials. Their initial hypothesis postulated that pretreatment methods such as sandblasting and silica coating might exert an influence on the bonding strength. However, the research findings contradicted this hypothesis. Instead, they explored three distinct approaches for pretreating In-Ceram alumina: airborne particle abrasion, tribochemical silica coating, and nano-structured alumina coating. Notably, the outcomes of their study demonstrated that the nano-structured alumina coating method yielded the highest bonding strength, signifying a robust interconnection between the materials. In contrast, both the airborne particle abrasion and tribochemical silica coating techniques exhibited a higher incidence of bond strength failures [83]. In a separate study conducted by Abousheilib et al. [84], a different approach was recommended. Their research suggested the use of selective infiltration etching, which was found to be more effective than sandblasting and silica coating. This method resulted in less structural loss and, consequently, higher bond strength values [85].

### 5.3. Coloring Shades

Restorative materials utilized in dental procedures often necessitate color adjustments to align with aesthetic standards. Oxides such as ferric oxide (Fe_2_O_3_) and cerium oxide (CeO_2_) not only contribute to the coloration of yttria-stabilized tetragonal zirconia polycrystals (Y-TZP) but can also influence their thickness. However, these coloration processes may also impact the adhesive bond strength between the ceramic and resin components. Previous research studies have involved immersing zirconia materials in various coloring agents denoted as B2, C1, D4, and A3 shades. The outcomes of these studies have revealed divergent effects on the bond strength of zirconia ceramic when paired with Panavia V5 cement. Specifically, it was observed that the A3 shade coloring did not exert a significant influence on the bond strength. In contrast, the other three types of colorings, namely B2, C1, and D4 shades, exhibited distinctive effects. Both B2 and C1 shade colorings were found to cause a linear reduction in the bond strength between the ceramic and resin. Furthermore, the magnitude of this adverse impact was directly proportional to the concentration of the colorings used. It is noteworthy that the bond strength is influenced by two primary factors: the composition of the coloring agent and the microstructure of the zirconia surface. In contrast, the D4 shade coloring agent resulted in an increase in the bond strength between the ceramic and resin, showcasing a unique effect in comparison to the other studied colorings [85].

## 6. Conclusions

Achieving optimal bond strength in dental procedures hinges on meticulous preparation techniques, efficient MMP management, rigorous contamination control, and deliberate shade selection. These factors together play a pivotal role in ensuring the success and long-lasting durability of dental restorations. In summary, the following key conclusions can be drawn:

1. The micro test measurements are crucial for assessing bond strength in dental procedures, but they are underutilized due to their labor-intensive nature and sensitivity to technique.
2. The condition of the smear layer on dentin significantly influences bond strength, emphasizing the importance of maintaining the proper phosphoric acid concentration during preparation and selecting appropriate bur types, such as superfine-grit or diamond burs.
3. MMPs, particularly MMP-2 and MMP-9, pose challenges to dentin-resin bonding, with peak activity within the pH range of 2.3 to 4.5. The integration of MMP inhibitors into adhesive systems can effectively prevent hybrid layer degradation.
4. Contaminants like blood and saliva can impact resin bonding, and effective remedies include thorough flushing with water and the adoption of wet bonding techniques involving ethanol and acetone.
5. In the context of indirect restorations, ceromers are preferred for their superior wear resistance and bond strength compared to resin nanoceramics. Shade selection is also crucial, with A3 having minimal impact on bond strength, while shades B2 and C1 exhibit a linear decrease, and D4 contributes to an increase in bond strength.
